# Nanoscale switch for vortex polarization mediated by Bloch core formation in magnetic hybrid systems

**DOI:** 10.1038/ncomms8836

**Published:** 2015-08-04

**Authors:** Phillip Wohlhüter, Matthew Thomas Bryan, Peter Warnicke, Sebastian Gliga, Stephanie Elizabeth Stevenson, Georg Heldt, Lalita Saharan, Anna Kinga Suszka, Christoforos Moutafis, Rajesh Vilas Chopdekar, Jörg Raabe, Thomas Thomson, Gino Hrkac, Laura Jane Heyderman

**Affiliations:** 1 Laboratory for Mesoscopic Systems, Department of Materials, ETH Zurich, 8093 Zurich, Switzerland; 2 Paul Scherrer Institute, 5232 Villigen PSI, Switzerland; 3 Department of Materials Science and Engineering, University of Sheffield, Sheffield S1 3JD, UK; 4 College of Engineering, Mathematics and Physical Sciences, University of Exeter, Exeter, Devon EX4 4SB, UK; 5 School of Computer Science, University of Manchester, Manchester M13 9PL, UK

## Abstract

Vortices are fundamental magnetic topological structures characterized by a curling magnetization around a highly stable nanometric core. The control of the polarization of this core and its gyration is key to the utilization of vortices in technological applications. So far polarization control has been achieved in single-material structures using magnetic fields, spin-polarized currents or spin waves. Here we demonstrate local control of the vortex core orientation in hybrid structures where the vortex in an in-plane Permalloy film coexists with out-of-plane maze domains in a Co/Pd multilayer. The vortex core reverses its polarization on crossing a maze domain boundary. This reversal is mediated by a pair of magnetic singularities, known as Bloch points, and leads to the transient formation of a three-dimensional magnetization structure: a Bloch core. The interaction between vortex and domain wall thus acts as a nanoscale switch for the vortex core polarization.

## Abstract (web summary)

Magnetic vortices in thin ferromagnetic films possess a core with out-of-plane magnetization whose polarity can be manipulated by magnetic fields or currents for technological applications. Here, the authors demonstrate local control of the core polarity in NiFe films via an imprinted maze domain pattern.

## Graphical abstract

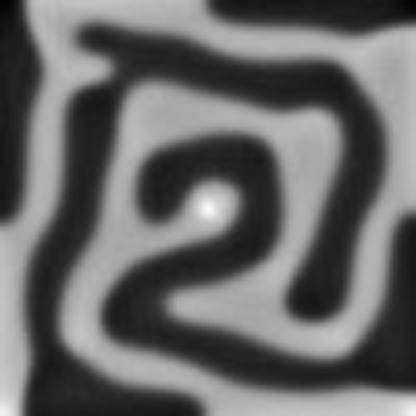

In recent years, the study of magnetic vortices has evolved into a large and intense field of research due to the fact that vortices are fundamental magnetic structures that hold promise for future technological applications such as information carriers, microwave sources or magnetic sensors. Vortices consist of a stable magnetic flux-closure domain state characterized by an in-plane curling of the magnetization around a very stable and narrow central core, ∼10 nm in diameter, magnetized out-of-plane^1^,^2^. While the bistability of the vortex core (with magnetization pointing up or down) is appealing for novel non-volatile memory storage concepts^3^,^4^, the dynamics of vortices is also of interest for applications such as radio frequency oscillators^5^,^6^, logic devices^7^,^8^ and magnonic crystals^9^,^10^, where the gyrotropic motion^11^ of the vortex core is exploited.

The key to a reliable implementation of vortices for such applications is control of the vortex core orientation. Previous work has shown that the vortex core polarization can be reversed^12^ and selected^13^ using in-plane alternating magnetic fields as well as ultrashort field pulses^14^, spin-polarized currents^15^,^16^ and spin waves^17^,^18^. Here we demonstrate a route to locally control the vortex core polarization in a hybrid magnetic structure, which combines two magnetic films with orthogonal anisotropies, leading to a mutual imprinting of the vortex and maze domain states, and therefore new degrees of freedom in the magnetization dynamics. We show that the vortex core switching occurs in the in-plane layer of a patterned element at the maze domain boundary and is driven by the underlying perpendicular domain state (schematically illustrated in Fig. 1). In our realization, the in-plane material is a Permalloy (Ni_80_Fe_20_) layer in a flux-closure state, while the perpendicular material is a [Co(0.3 nm)/Pd(0.9 nm)]_8_ multilayer. Using micromagnetic simulations, we elucidate the influence of the magnetic domains in the Co/Pd multilayer on the vortex core dynamics and show that the reversal is a complex three-dimensional process mediated by the formation of a magnetic discontinuity that connects two Bloch points, which we call a Bloch core. This is a fundamentally different mechanism compared with reversal in single-material structures that results from the complex magnetic configuration. The mutual interaction with a maze domain wall provides a highly localized switch allowing control of the magnetization at the nanoscale.

## Results

### Scanning transmission X-ray microscopy measurements

**Figure 2:**
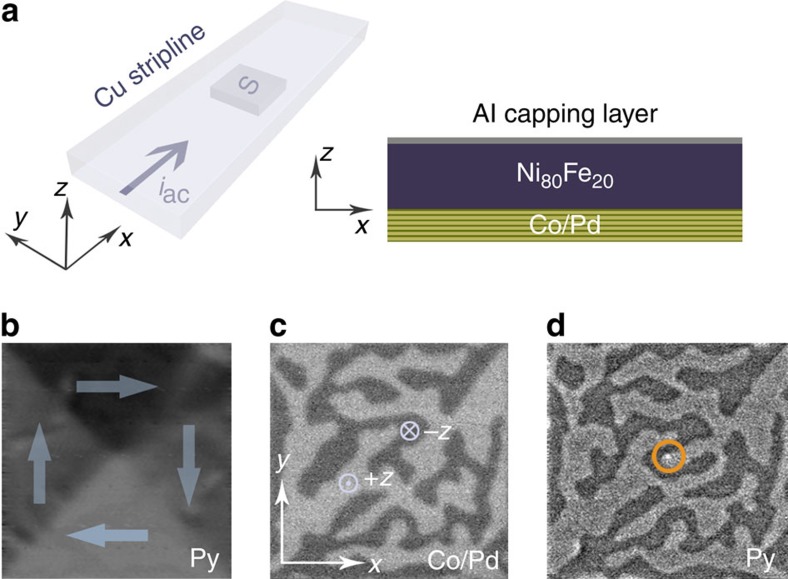
Sample geometry and static XMCD images of magnetic domain configurations. (**a**) Schematic diagram of the patterned multilayer square (S) embedded in the stripline, showing the direction of the applied current, *i*_*ac*_ and the coordinate convention used as well as the layer stack. The interface between the Py (Ni_80_Fe_20_) and the Co/Pd layers is defined as *z*=0 nm. (**b**) Typical in-plane Landau pattern measured in a 50 nm thick Py layer of a 3 × 3 μm square (30°, Ni *L*_3_ edge). The arrows indicate the direction of the local magnetization. (**c**) Out-of-plane maze domain state in the [Co(0.3 nm)/Pd(0.9 nm)]_8_ multilayer of a similar 3 μm × 3 μm square (normal incidence, Co *L*_3_ edge) on the same sample. The direction of the out-of-plane magnetization is indicated. (**d**) Imprinted out-of-plane maze domain state in the Py layer with the vortex core visible as a white spot in the centre of the structure (highlighted by circle; normal incidence, Ni *L*_3_ edge). Images **c** and **d** are taken from the same square. Image **d** was taken after the application of static magnetic fields large enough to move the vortex core and slightly alter the domain configuration as a result of the mutual interaction between the Py and the Co/Pd layers. The contrast of each image is adjusted for better visibility.

To investigate the dynamics in the hybrid system, we fabricated square thin film structures consisting of a 50 nm thick Permalloy (Py) layer with 3 μm and 5 μm side length deposited on top of a Co/Pd multilayer (Fig. 2a). The structures were imaged using scanning transmission X-ray microscopy (STXM)^19^ exploiting the X-ray magnetic circular dichroism (XMCD) effect^20^, which allows for element-specific imaging of the in-plane and out-of-plane magnetization depending on the orientation of the sample with respect to the polarization vector of the X-ray beam. On probing the in-plane component of the magnetization and tuning the X-ray energy to the Ni absorption edge, the flux-closure Landau state in the Py is imaged (Fig. 2b; Supplementary Fig. 1; for experimental details see Methods). When the X-ray energy is tuned to the Co absorption edge, the maze domains in the Co/Pd multilayer are observed (Fig. 2c), demonstrating how the interaction between the Py and Co/Pd causes the Co/Pd domain walls to preferentially align along the Néel walls in the Py layer. Imaging the out-of-plane component of the magnetization of the Py layer reveals the vortex core and the presence of an imprinted maze domain state (Fig. 2d). The vortex core is found close to the maze domain boundary rather than in the centre of the domain, therefore minimizing the energy associated with the stray field of the vortex core (∼200 mT in a single-layer Py film)^14^. The imprinting of the maze domains in the Py layer is a result of the exchange and magnetostatic interactions between the Py layer and the Co/Pd multilayer stack^21^. In the Py, the vortex core polarization is identical to the magnetization orientation in the underlying, imprinted (perpendicular) maze domain.

**Figure 3:**
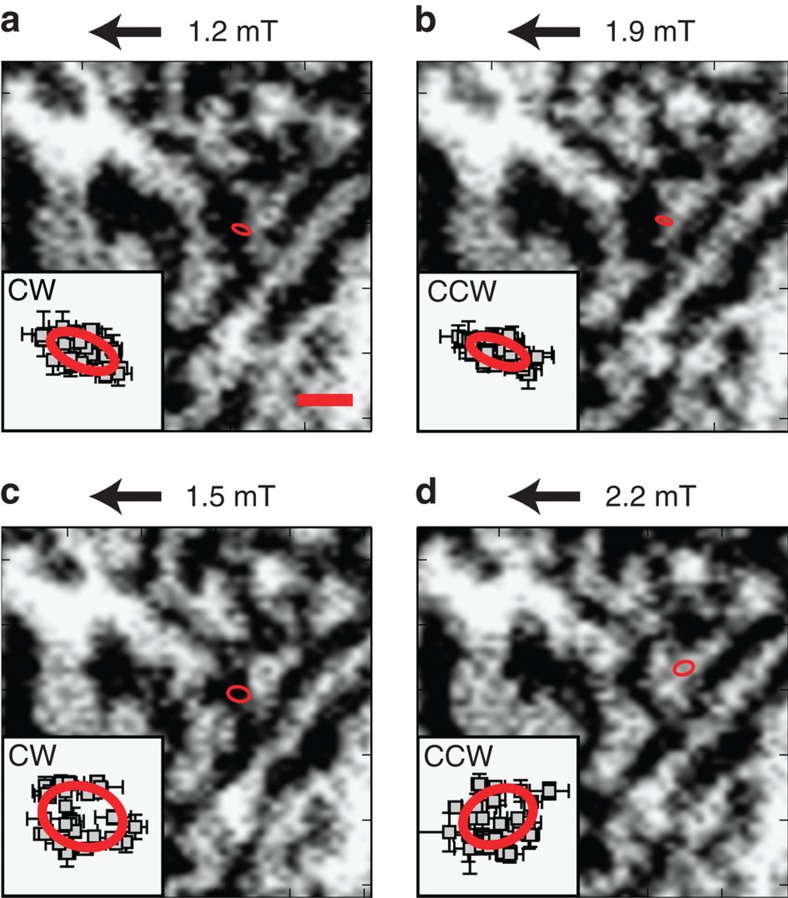
Vortex core trajectories in Py layer overlaid on Co/Pd maze domain configuration. Maze domain state of the Co/Pd multilayer with out-of-plane magnetization in a 5 μm × 5 μm square measured by XMCD at the Co *L*_3_ edge. The vortex core in the adjacent Py layer with in-plane magnetization is excited with a 258 MHz a.c. field of amplitude 3.9 mT and enters a state of dynamic motion marked by the elliptical fit in each panel. The sense of gyration of this motion, clockwise (CW) or counterclockwise (CCW), is used as a probe of the polarization of the vortex core. By varying a static magnetic field (in the direction indicated by arrows), the vortex core is displaced laterally (**a**–**d**) and reversal of the sense of gyration of the vortex core is observed. The insets show the vortex core positions during the core gyration together with an elliptical fit of 31 vortex core positions. The bars indicate the error resulting from the detection of the vortex core position in each scan. The scale bar is 500 nm and the field of view of the insets is 320 nm × 320 nm. The bright (dark) contrast corresponds to the magnetization pointing out of (into) the plane and is adjusted for better visibility.

To study the influence of the maze domains on the dynamics of the Landau state, the gyrotropic motion of the vortex core is excited using an alternating magnetic field created by injecting a sinusoidal alternating current (a.c.) into a Cu stripline fabricated on top of the magnetic structures. On application of an additional static magnetic field, the vortex core can be displaced and its lateral position controlled during the gyration. The polarization of the vortex core is probed by detecting the sense of the vortex gyration^22^, which is determined by the gyrocoupling vector^23^ **G**=(2*πμ*_0_*M*_*s*_*h*/*γ*_0_*)np***z**, where *n* is the vortex winding number (*n=1*), *p* the vortex core polarization (*p*=±1), *h* the sample thickness, *M*_*s*_ the saturation magnetization, *μ*_0_ the vacuum permeability and *γ*_0_ the gyromagnetic ratio. The fitted trajectories of the vortex core motion in the Py layer of a 5 μm × 5 μm structure under application of different static magnetic fields of 1.2 mT (a), 1.9 mT (b), 1.5 mT (c) and 2.2 mT (d) are shown in Fig. 3. These trajectories are overlaid on the corresponding images of the maze domain state in the Co/Pd multilayer. In a static 1.2 mT field (Fig. 3a), the vortex core gyrates clockwise (see Supplementary Movie 1, Supplementary Note 1 and Supplementary Fig. 2) in a maze domain displaying dark contrast, which indicates an out-of-plane magnetization in the -*z* direction. An increase in static field strength to 1.9 mT (Fig. 3b) displaces the vortex core so that it gyrates in a bright domain within the error bars shown in the inset. At the same time, the sense of gyration has reversed (counterclockwise), indicating that the vortex core polarization has switched (+*z* direction). Decreasing the static field to 1.5 mT returns the vortex core to the dark region (Fig. 3c) and the sense of gyration is again reversed (clockwise). A subsequent increase of the static field to 2.2 mT shifts the vortex core into a bright region and a gyration in the opposite direction (counterclockwise) is observed (Fig. 3d and Supplementary Movie 1). Our results indicate that the vortex core trajectories are confined by the maze domains and that the polarization of the gyrating vortex core is aligned with the magnetization in the underlying domain. The vortex core reversal must therefore occur as it crosses the boundary between maze domains. Noticeable changes in the maze domain state are also apparent in the sample, particularly in the vicinity of the vortex core trajectory. This is seen, for example, in Fig. 3d where the bright domain, in which the core is located, expands at the expense of the dark domain that previously contained the core (Fig. 3c). Such changes are due to the interaction between the vortex core and the maze domain wall during the vortex dynamics driven by an external a.c. field.

### Micromagnetic simulations

**Figure 4:**
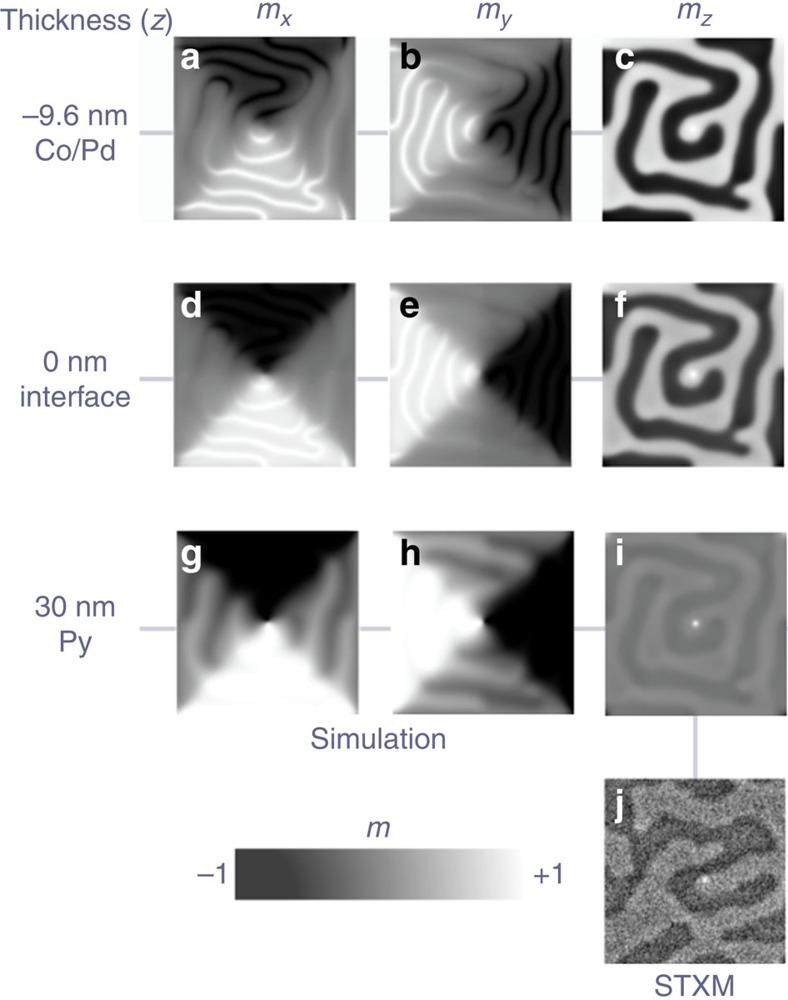
Depth-dependent simulated magnetic configurations in the [Co/Pd]/Py hybrid system. (**a**–**i**) Simulated *m*_*x*_, *m*_*y*_ and *m*_*z*_ components of the initial remanent magnetization states at different thicknesses *z* in the hybrid structure (*z*=−9.6 nm is the bottom of the Co/Pd multilayer stack, *z*=0 the interface between the Co/Pd and the Py layers and *z*=30 nm is the top of the Py layer). In the simulations, the side length of the square is 800 nm and the Py layer is 30 nm thick. In **i**, the simulated out-of-plane component of the magnetization state at the top of the sample (Py layer) displays similar features to those experimentally observed in **j**, which is an XMCD image of the central 1.5 μm × 1.5 μm region of the 3 μm × 3 μm square, taken at normal incidence at the Ni *L*_3_ edge.

To elucidate the details of this reversal mechanism, we have performed micromagnetic simulations of the vortex core dynamics as it crosses a Co/Pd maze domain boundary. In Fig. 4, we first show the static magnetic structure obtained from the simulations, which confirms the experimentally observed mutual imprint of domain configurations in the Co/Pd and Py layers. The maze domain state in the Co/Pd multilayer is modified by the Landau state in the Py layer as shown in Fig. 4a,b. Here it can be seen that the internal in-plane magnetization (*m*_*x*_ and *m*_*y*_) of the maze domain walls follows the magnetization orientation in the Py film, so that the maze domain walls bend at the location of the Néel walls associated with the Landau state in the Py. The out-of-plane magnetization (*m*_*z*_) is shown in Fig. 4c. At the interface (*z*=0) between the Co/Pd and the Py (Fig. 4d–f) the mutual imprint is strongest. The different magnetic anisotropies of the coupled Co/Pd and Py layers result in strong depth-dependent magnetization so that the maze domain state is more prominent in the Co/Pd multilayer and the Landau state is more prominent in the Py layer (Fig. 4g–i), but both extend throughout the entire magnetic structure thickness. The vortex core, which is also imprinted in the Co/Pd multilayer, has a polarization that matches the orientation of the underlying maze domain structure (Fig. 4c,f,i). In an isolated Py layer of the same thickness, the core has a non-uniform structure that is wider in the interior of the film and narrower towards the surfaces^24^ and may give rise to flexure modes^25^. However, in our sample, the simulations show that there is little bending of the core structure along the Py thickness as a result of the coupling with the out-of-plane Co/Pd layer. In Fig. 4j, an experimental image of an equivalent configuration is given for comparison with the simulations. The maze domain pattern is determined by the interplay between anisotropy, exchange and magnetostatic energies and can therefore be controlled by the Co/Pd anisotropy, layer thickness and sample geometry.

**Figure 5:**
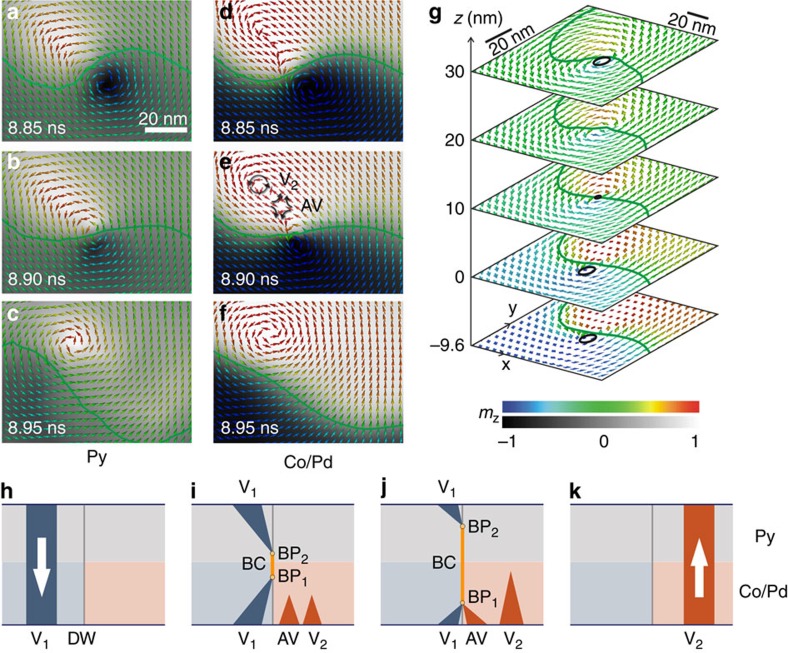
Modelled Bloch core reversal. (**a**–**f**) Magnetization dynamics in the Py and Co/Pd layers, respectively, 10 nm above (**a**–**c**) and 9.6 nm below the interface (**d**–**f**), during vortex core reversal. The green line indicates the centre of the maze domain wall where *m*_*z*_=0. The sequences show the vortex core reversing its polarization (dark to bright *m*_*z*_ contrast) as it crosses the maze domain wall. The initial bright contrast in **a** corresponds to the region with out-of-plane magnetization that forms during the vortex core motion. In the Co/Pd multilayer, a vortex-antivortex pair is created during the reversal process (**e**). Thickness-dependent cross-sections of the magnetic structure during the vortex core reversal are shown in **g** at 8.90 ns, highlighting the vortex core (circles) and the domain wall (green line). While the vortex core radius is finite at the top and bottom surfaces, it vanishes within the sample where the magnetization is purely in-plane. Note that the plotted arrow density is lower than in **a**–**f** for clarity. (**h**–**k**) Schematic representation of the time evolution of the out-of-plane component of the magnetization during the vortex core reversal via the formation of a Bloch core (BC, orange line) at a domain wall (DW) separating two oppositely magnetized out-of-plane domains (pale blue and red regions). In **i** and **j**, a new vortex–antivortex pair (V_2_–AV) is created in the Co/Pd multilayer with opposite polarization compared to the original vortex core (V_1_). As the Bloch point BP_1_ moves towards the bottom surface, it mediates the annihilation of the original vortex core V_1_ with the antivortex, AV, in the Co/Pd multilayer. The initial vortex core V_1_ is dissolved in the Py as BP_2_ reaches the top surface. The grey scale concerns images **a**–**f** and represents the orientation of the out-of-plane magnetization. The colour scale represents the out-of-plane orientation of the magnetization associated with the arrows in **a**–**g**, which are plotted in the (*x*,*y*) plane for clarity. In **g**, the arrows are plotted with full 3D orientation. Hence, the curl around the vortex and antivortex in **e** is less obvious in **g** due to the strong out-of-plane component. The simulated square has an edge length of 800 nm and the Py layer is 30 nm thick.

Starting from the remanent state, an a.c. current matching the experimental parameters is applied in the simulation to probe the dynamics of the system. The resulting Oersted field causes the vortex core to gyrate and, as the vortex is excited, it enters a spiral trajectory and a region with a strong out-of-plane component with opposite magnetization develops in the vicinity of the vortex core in the Py layer (bright region adjacent to the dark region delimiting the vortex core in Fig. 5a). Once the vortex core reaches an equilibrium radius, it describes a circular motion and reverses its polarization as it crosses from one maze domain to another oppositely magnetized domain, passing across a domain wall with purely in-plane magnetization at its centre (Fig. 5a-f, centre of wall position indicated with solid green line). As the initial vortex core (*V*_1_ in Fig. 5h) approaches the maze domain wall, its internal structure is distorted through the sample thickness, such that the central section of the vortex core (at the interface between the Py and Co/Pd) merges with the wall before the top (*z*=30 nm) and bottom (*z*=−9.6 nm; see Fig. 5g, which represents the same moment in time as Fig. 5b,e,i). When the vortex core (V_1_) coincides with the domain wall, its structure is dissolved by the creation of a pair of magnetic singularities, or Bloch points (BP_1_ and BP_2_ in Fig. 5i). Such singularities occur when the magnetization cannot continuously unwind, resulting in a region of locally diverging magnetization over a few nanometers^26^,^27^. The extended region joining the Bloch points is characterized by a line singularity extending through the sample thickness. We refer to this new structure as a Bloch core (orange line in Fig. 5i,j). The Bloch core is topologically related to the magnetic drops reported to occur during magnetization reversal in cylindrical nanowires^28^. Due to the strong out-of-plane anisotropy in the Co/Pd multilayer, a vortex–antivortex pair (AV and V_2_ in Fig. 5i) nucleates in the vicinity of the original vortex with polarization opposing the original vortex core (Fig. 5e). The reversal of the vortex core polarization begins with the annihilation of the original vortex core (V_1_) and the antivortex (AV) at the domain wall in the Co/Pd (Fig. 5j). This annihilation is mediated by one Bloch point (BP_1_) at the boundary of the Bloch core. The vortex core polarization reversal is complete when the new vortex core (V_2_) expands and the remaining Bloch point (BP_2_) is expelled at the surface of the Py layer (Fig. 5j,k). Despite the complexity of this process, the reversal itself takes place within less than 100 ps. Excess energy generated by the polarization reversal is dissipated in the form of spin waves^29^. It should be noted that the reversal occurs once a threshold corresponding to the formation energy of the new vortex–antivortex pair is reached^30^. Hence, the reversal requires an a.c. excitation. The simulations also confirm that the vortex gyration and the reversal of the core polarization result in local changes to the maze domain structure in the proximity of the vortex core (see Supplementary Movie 2 and Supplementary Note 2). In addition, the maze domain wall magnetization is locally reversed as the vortex core crosses it, since its magnetization is defined by the vortex chirality. This change in the wall structure preserves the time reversal symmetry during the switching process.

In summary, we have shown that the interaction between the out-of-plane vortex core magnetization in a Landau state and a maze domain wall with local in-plane magnetization results in a fast (∼100 ps) and highly localized switch for the vortex polarization. Moreover, the vortex core reversal mechanism is distinct from its counterpart in homogeneous structures, which occurs through the creation and annihilation of a vortex-antivortex pair^12^,^14^ or by punch-through^31^, that involve the nucleation of a single Bloch point (see Supplementary Fig. 3). In our hybrid system, the vortex polarization reversal, mediated by a Bloch core and characterized by the formation of a pair of singularities, leads to the temporary suppression of the vortex core magnetization at the maze domain wall. The possibility to switch the core polarization at a specific location is of interest for a variety of low power applications. For example, this added functionality could be used to trap and release labelled magnetic nanoparticles with the vortex core stray field. Moreover, the trapped particles could be identified through detection of the vortex gyration frequency, which will be modified depending on the material and size of the trapped particle. Further possibilities include tuneable RF oscillators and dynamic encryption devices, with the out-of-plane domain pattern controlled through the sample geometry or through lithographic engineering of the Co/Pd multilayer.

## Methods

### Sample fabrication

The [Co/Pd]/Py bilayer square structures with side lengths of 3.0 μm and 5.0 μm were fabricated on a silicon nitride membrane using electron beam lithography and lift-off processing. The magnetic layer stack is [Co(0.3 nm)/Pd(0.9 nm)]_8_/Pd(1.5 nm)/Ni_80_Fe_20_(50 nm) with a seed layer of Ta(1.5 nm)/Pd(1.6 nm) and a 1 nm Al cap to prevent rapid oxidation. We use a 1.5 nm thick Pd spacing layer between the Co/Pd and the Py, thus reducing the coupling (exchange and magnetostatic) between the layers and increasing the vortex core mobility. The stripline consisting of Ti(5 nm)/Cu(200 nm)/Ti(5 nm) is patterned on top of the structures. The whole sample is covered by 150 nm AlN to dissipate the Joule heating created by the current flowing through the stripline. All materials were deposited by d.c. magnetron sputtering at normal incidence without rotation at room temperature. The base pressure was 10^−6^ mbar and the Ar pressure 2 × 10^−3^ mbar.

### Scanning transmission X-ray microscopy

The images were taken using scanning transmission X-ray microscopy (STXM)^19^ exploiting X-ray magnetic circular dichroism (XMCD)^20^. The contrast depends on the relative orientation of the sample magnetization and the X-ray polarization vector, being maximum for parallel and minimum for antiparallel alignment, with intermediate contrast representing magnetization orthogonal to the X-ray polarization vector. To obtain XMCD images, we divide two images that are taken with opposite helicities.

The static images were taken both with a 30° angle of incidence and at normal incidence of the X-ray beam to probe the in-plane and out-of-plane components of the magnetization, respectively. To investigate the magnetic state of the individual layers, images were taken at the Co *L*_3_ absorption edge for the Co/Pd multilayer and at the Ni *L*_3_ absorption edge for the Py layer.

The dynamic images were recorded at the Ni *L*_3_ absorption edge with the sample rotated about the *y* axis giving an angle of 30° between the surface normal and the direction of the X-ray propagation (+*z* direction in Fig. 2) to be mainly sensitive to the in-plane component of the magnetization in the Py layer. Corresponding images of the Co/Pd maze domain configuration were taken at the Co L_3_ absorption edge. In Fig. 3, the images have been taken using a single X-ray helicity, which gives sufficient contrast. The alternating magnetic field was generated by applying an a.c. current (258 MHz, 62 mA) through the Cu stripline creating a magnetic field with amplitude 3.9 mT.

The elliptical fits of the vortex core trajectories were performed using the least squares method. The long/short axes of the fitted ellipses in Fig. 3 are: (a) 136 nm/64 nm, (b) 117 nm/49 nm, (c) 164 nm/120 nm, (d) 146 nm/106 nm with an error of <44 nm for the long axes and <32 nm for the short axes. The positions of the fitted trajectories of the vortex core in the Py were superimposed onto the perpendicular maze domains in the Co/Pd by matching the edges of the patterned squares in the respective measurements. The uncertainty in the determination of this offset is <19 nm. The sense of the vortex core gyration can be directly determined from the dynamic images (see Supplementary Movie 1). The positions of the vortex core during the motion were obtained by detecting the maximum change in contrast. The experimental lateral resolution given by the beam spot size was ∼70 nm.

### Micromagnetic model

We solved the Landau–Lifshitz–Gilbert equation for multilayer squares of side length 0.8 to 1.2 μm and a Py thickness of 30 nm (Fig. 2a) using finite element modelling^32^. The interlayer exchange between the Co/Pd and the Py is modelled using an intergrain exchange model^33^ including the Oersted field. The element edge size is adaptively increased from 4 nm at the centre of the square to 12 nm at the edges. The magnetic material was surrounded by Cu (10 nm) on the top and side surfaces, perpendicular to the *x* axis. We modelled the current as a triangular wave of amplitude 36 mA (peak Oersted field 7.4 mT), matched to the experimental frequency. Other effects due to the current, such as Ohmic heating and spin-polarization, have been simulated but were found not to be essential to the vortex core reversal process.

We treated the Co/Pd multilayer as a single material with electrical conductivity *σ*_Co/Pd_=3 MSm^−1^, exchange stiffness *A*_Co/Pd_=10 pJ m^−1^, saturation magnetization *M*_Co/Pd_=400 kA m^−1^, perpendicular anisotropy *K*_Co/Pd_=250 kJ m^−3^ and damping constant *α*_Co/Pd_=0.02. The Py parameters were *σ*_NiFe_=3 MSm^−1^, *A*_NiFe_=13 pJ m^−1^, *M*_NiFe_=800 kA m^−1^, *K*_NiFe_=0 kJ m^−3^ and *α*_NiFe_=0.02. The conductivity of Cu was *σ*_Cu_=4.5 MSm^−1^. The smaller size of the simulated structures was chosen to reduce computation time and, at the same time, to adequately reproduce the observed magnetic configurations and their dynamics.

## Additional information

**How to cite this article:** Wohlhüter, P. *et al.* Nanoscale switch for vortex polarization mediated by Bloch core formation in magnetic hybrid systems. *Nat. Commun.* 6:7836 doi: 10.1038/ncomms8836 (2015).

## Supplementary Material

Supplementary Information Supplementary Figures 1-3, Supplementary Notes 1-2, and Supplementary References

Supplementary Movie 1 Reversal of the vortex gyration direction measured with STXM at two different applied fields corresponding to Fig. 3a and 3d. Each movie consists of 31 images obtained during a gyration cycle.

Supplementary Movie 2 Simulation of the vortex core reversal in the presence of an AC field in an 800 nm × 800 nm square with the contrast corresponding to the out-of-plane component of the magnetization. The time evolution of the applied AC field is shown in the inset.

## Figures and Tables

**Figure 1:**
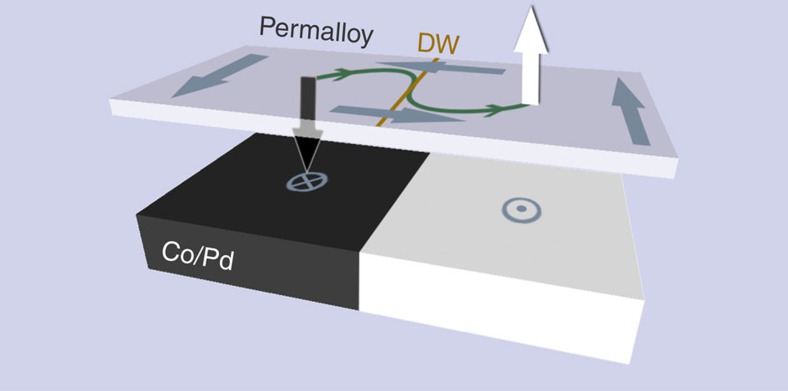
Schematic diagram of vortex core reversal process. The magnetization in a resonantly excited vortex core (black out-of-plane arrow) reverses its orientation as it crosses and interacts with a domain wall (DW) at the boundary between two adjacent, oppositely oriented out-of-plane maze domains originating in the Co/Pd multilayer (bright and dark domains). The path followed by the excited vortex core is illustrated in green: the vortex core (black arrow) initially precesses clockwise, and then counterclockwise after reversing its polarization (white arrow) at the domain boundary (orange line). The in-plane magnetization in the Py layer is illustrated by the blue arrows.
