# Supplementary material for: Nanoscale switch for vortex polarization mediated by Bloch core formation in magnetic hybrid systems
Source: Nat Commun. 2015 Aug 4;6:7836. doi: 10.1038/ncomms8836 (PMC4532796; doi:10.1038/ncomms8836)
Supplement: Supplementary Information — Supplementary Figures 1-3, Supplementary Notes 1-2, and Supplementary References [file ncomms8836-s1.pdf]

Supplementary Figure 1:

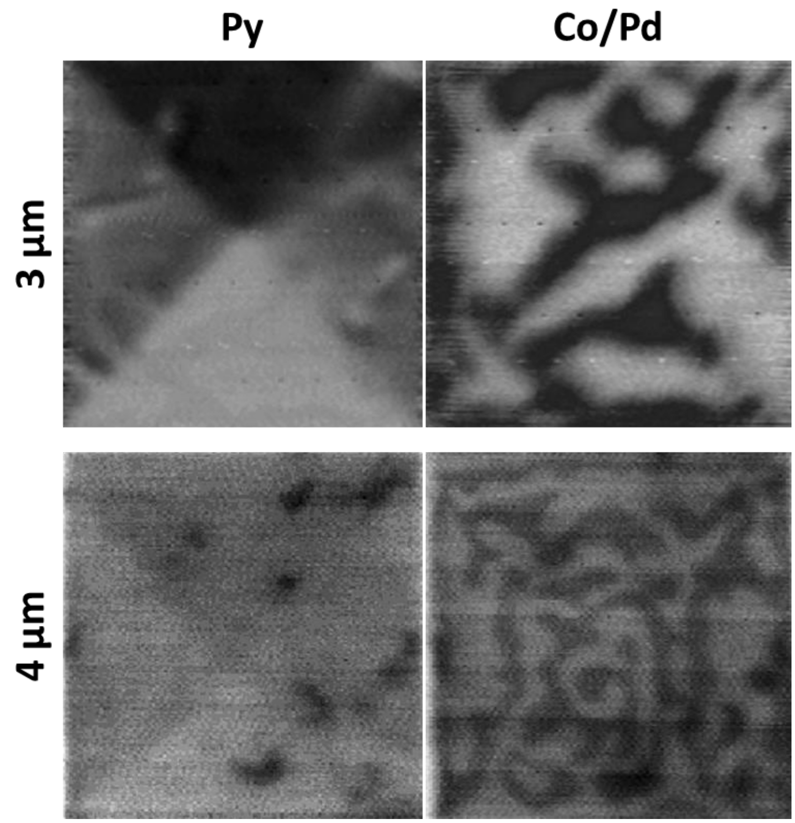

Domain configuration of the Co/Pd and Py layers in 3 µm and 4 µm squares. In the 3 µm square, the magnetic configuration in the Co/Pd layer and the corresponding Landau state from Fig. 2b is shown, as well as additional corresponding Co/Pd and Py domain configurations in a 4 µm square. The configurations of the maze domains in the Co/Pd are modified by the Landau state in the neighbouring Py layer. The images were taken at an angle of 30° at the Ni and Co L_3 absorption edges for the Py and Co/Pd layers, respectively.

Supplementary Figure 2:

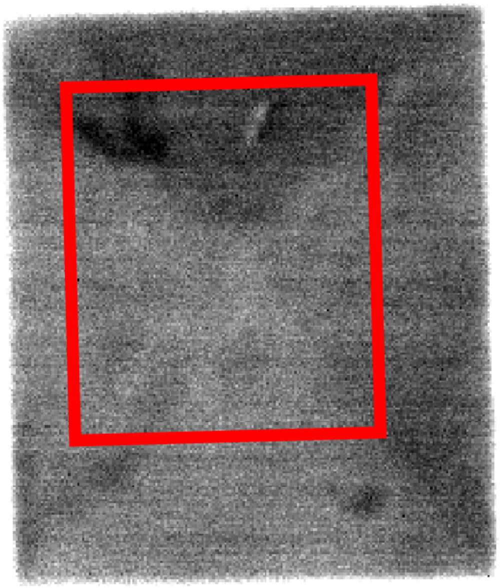

Field of view in Fig. 3. First frame of the left Supplementary Movie showing the entire 5.0 µm × 5.0 µm square in a Landau state. The red box indicates the field of view displayed in Fig 3. The image was taken at 30° orientation at the Ni L_3 absorption edge and has not been corrected for this tilt.

Supplementary Figure 3:

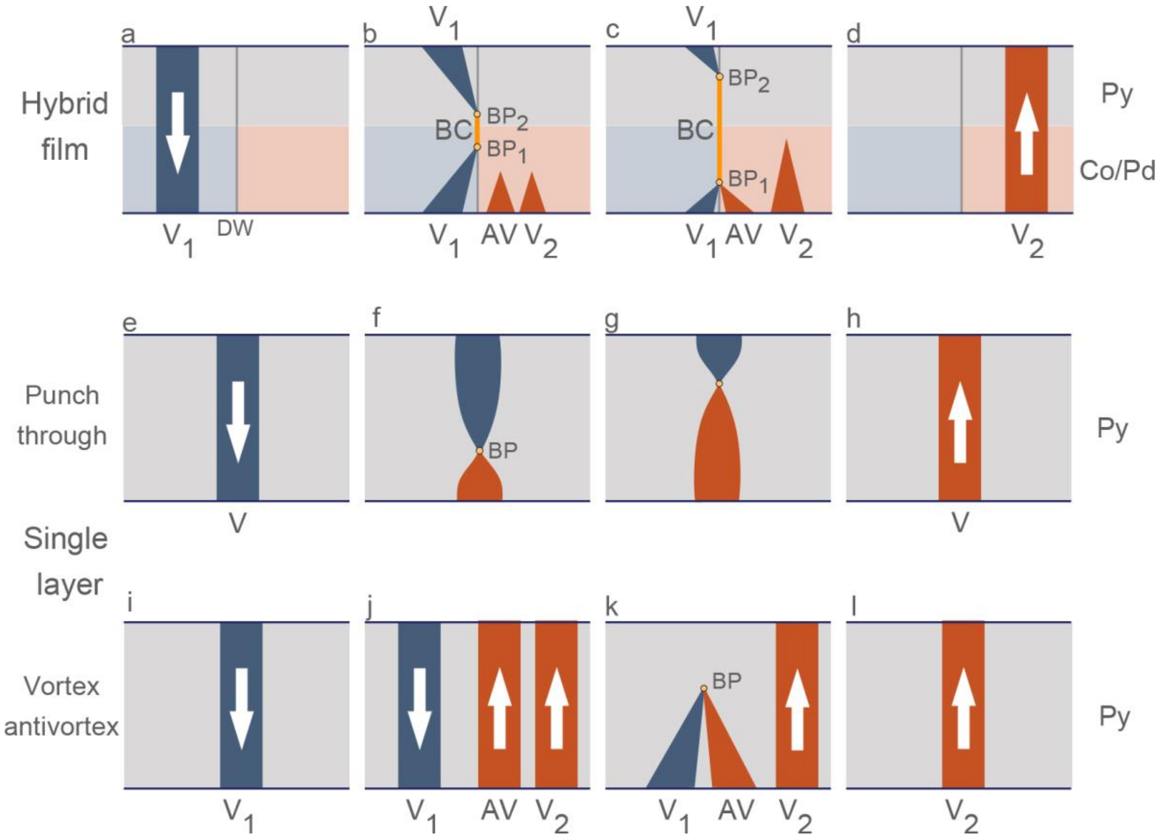

Schematic diagrams of various vortex core reversal mechanisms. The time evolution of the out-of-plane component of the magnetization during the vortex core reversal via Bloch core formation in a hybrid film (a-d), as well as punch-through (e-h) and vortex-antivortex nucleation (i-l) in single material films are shown.

(a-d): In the hybrid film, the vortex core (V_1) is dissolved at a domain wall (DW) separating two oppositely magnetized out-of-plane domains (blue and red regions), resulting in the formation of a Bloch core (BC) containing a line singularity. In (b), a new vortex-antivortex pair (V_2-AV) is created in the Co/Pd film with opposite polarization compared to the original vortex core (V_1). No pair creation occurs in the Py film. As the Bloch point BP_1 moves towards the bottom surface, it mediates the annihilation of the original vortex core V_1 with the antivortex in the Co/Pd layer. The initial vortex core V_1 is dissolved in the Py by the motion of the second Bloch point, BP_2, to the upper surface.

(e-h): In single material films, the core can be reversed by applying a static magnetic field in the opposite direction to the core polarization. This 'punch-through' reversal involves the injection of a Bloch point at one of the surfaces and its propagation through the sample.[1]

(i-l): Reversal via vortex-antivortex pair creation in single films. A new vortex-antivortex pair nucleates in the film, and a Bloch point injected at a surface mediates the annihilation of the initial vortex and the new antivortex.[2]

Supplementary Note 1:

Description of Supplementary Movie 1, which shows the reversal of the vortex gyration direction measured with STXM at two different applied fields. STXM data of the vortex core gyration. The vortex core is located at the intersection of the Néel walls, before (left) and after (right) it crosses a maze domain boundary. The switch of the vortex core direction results in a reversal of the sense of gyration (indicated by the red arrows). The STXM data correspond to Fig. 3a and Fig. 3d. Each movie consists of 31 images obtained during a gyration cycle. The magnetic AC field was always on during the experiment and a static magnetic field (amplitude indicated above the movie) is used to displace the vortex core. In Fig. 3, only a 3.1 µm × 3.1 µm region is shown, where the vortex core gyration takes place. This region is indicated in Supplementary Fig. 1. The movies were taken at 30° orientation at the Ni L_3 absorption edge and have not been corrected for this tilt. (In order to see the orbital motion of the vortex core, the movie should be looped.)

Supplementary Note 2:

Description of Supplementary Movie 2, which shows the simulation of the vortex core reversal in the presence of an AC field. Simulated vortex core reversal in an 800 nm × 800 nm square with the contrast corresponding to the out-of-plane component of the magnetization. The time evolution of the applied AC field is shown in the inset. The original vortex core (black dot), before the reversal, and its clockwise gyration are shown as well as the formation of an out-of-plane region in its vicinity. The reversal results in a sudden burst of spin waves. Following the reversal, the new vortex core (white dot) gyrates counterclockwise. The core influences the out-of-plane maze domains, locally modifying the domain pattern. Changes in the domain pattern also occur at the sample edges due to the Oersted field. Modifications of the domain structure are accompanied by the emission of spin waves.

Supplementary References

1. Thiaville A, Garcia JM, Dittrich R, Miltat J, Schrefl T. Micromagnetic study of Bloch-point-mediated vortex core reversal. *Phys Rev B* 2003, **67**(9)**:** 094410.

2. Hertel R, Gliga S, Fähnle M, Schneider CM. Ultrafast Nanomagnetic Toggle Switching of Vortex Cores. *Phys Rev Lett* 2007, **98**(11)**:** 117201.
